# On Pulsation in the Epigastrium as an Accompanying Symptom of Indigestion

**Published:** 1839-04

**Authors:** 


### Art. XII.

*Ueber die Pulsation in der Oberbauchgegend als begleitendes Symptom der Indigestion.* Von Dr. C. Hohnbaum.—*Hildburghausen*, 1836.
*On Pulsation in the Epigastrium as an accompanying Symptom of Indigestion.* By Dr. Charles Hohnbaum.—*Hildburghausen*, 1836. 8vo. pp. 98.

Dr. Hohnbaum's experience has convinced him that epigastric pulsation is of much more frequent occurrence than is usually imagined, and that it is always complicated with derangement of the digestive organs. With this there is usually connected the well-known morbid condition commonly denominated *abdominal plethora;* but whether as cause or effect our author seems undecided. In such a state of things, the existing derangement of the venous circulation within the abdomen is apt soon to be reflected on the arterial system, in proof of which he refers to the irregular determinations of blood to the head, chest, &c., which frequently ensue, the hands and feet all the while continuing unnaturally cold. The face will now be frequently suffused, the pulse quick, full, and irregular, especially after taking food, the sleep restless and disturbed by dreams; headach and dizziness are often complained of, the temper is altered, and there is a disinclination to all exertion. It is at this period that the epigastric pulsation, depending apparently on impeded abdominal circulation, ordinarily makes its appearance, being occasionally, though by no means constantly, connected with a perceptible swelling in the epigastrium.

The author himself, who seems to be of an eminently nervous temperament, and very hypochondriacal withal, was for thirteen years the subject of this affection, and a great portion of his pamphlet is occupied in detailing his own case. Purgatives, venesection, and low diet he found useless. Leeches afforded some temporary relief. To the aperient waters of Carlsbad he ascribes much benefit, a large proportion of which may, we think, fairly be set to the account of change of air and scene, together with freedom from the fatigue and anxieties of his usual professional avocations. His health, which was from boyhood up delicate, had been still further impaired by the seductive but injudicious practice of prosecuting his studies up to a late hour in the night, a season at which, though the ideas may flow with greater freeness, there is in nervous individuals such a natural tendency to febrile excitement as is obviously indicative of the necessity of repose. In every case of the disorder which has fallen under his notice, the subject of it, whatever may have been his rank in life, was of sedentary habits. He points out at considerable length the diagnosis between pulsations of the kind which it is his object to investigate and those depending on aneurism of the aorta; or on tumours or enlarged abdominal organs pressing upon this vessel. The pulsations which we are now considering are often more conspicuous, though less permanent, than those of aneurism; they commonly supervene at stated periods, and more especially some time after eating. He believes that temporary distention of the bowels with feculent matter or flatus may, by pressing on the great vessels, occasionally give rise to this phenomenon. We have ourselves known well-marked abdominal pulsation to cease instantly on the shifting of wind from one part of the intestines to another: and a somewhat similar observation in regard to the transmission of the heart's action was made by Laennec in his own person. Kreysig speaks of having often met with these abdominal pulsations in hypochondriacal and hysterical patients, in fevers, before critical and other hemorrhages, in cases of suppressed hemorrhoids and in amenorrhœa. Baillie alludes to their sudden cessation on the supervention of gout.

As to the proximate cause of this affection little is known with certainty. Albers, who has written so elaborately on abdominal pulsations, conceives it to depend on a morbid condition of the abdominal nerves, and Senac, Burns, and Hope take a similar view of it. Dr. Law supposes that both it and the bruit de soufflet, which so frequently accompanies it, may depend on a temporary narrowing of the aorta produced by some irregular action of the nerves of the cœliac plexus which here surround it. Kreysig attributes it to an exalted state of vitality in a limited portion of the aorta; and Laennec, who had convinced himself that the action of the arterial system was in some degree independent of that of the heart, held a similar opinion, ascribing the pulsation to a locally increased energy of contraction in the artery.

The prognosis according to Dr. H. is generally favorable. "In respect to the treatment, as the proximate cause of the affection appears to consist in a partial obstruction of the abdominal circulation, and consequent accumulation of blood in the larger vessels, more especially the aorta, the chief indication consists in the removal of such obstruction." We should be taking a very narrow view of the treatment, continues our author, were we to confine our attention to the single symptom of epigastric pulsation, and make no account of the other evidences of derangement of the digestive organs, and of plethora of the veins within the abdomen, or finally of the marked excitement in the arterial system. Proofs of the last two may indeed occasionally be absent, but never those of disorder of the stomach or bowels; as, for example, flatulence, a sense of oppression and weight in the epigastrium, acidity, pyrosis; or else constipation or diarrhœa; or finally, a sense of anxiety, impeded respiration, hypochondriacism, melancholy, and distate to existence. When the epigastric pulsation makes its appearance in consequence of suppression of the menses or of any other accustomed evacuation, local or general bloodletting is indispensable; so also when there exists obvious excitement of the arterial system, or symptoms of congestion in the head or chest. By itself alone, however, epigastric pulsation rarely requires this remedy, and in the few cases where it may appear to be indicated leeches will generally be found to be more effectual than venesection. Warm footbaths are to be employed when the feet are habitually cold. Digitalis is decidedly injurious, as it tends still further to derange the digestive organs. The aperient neutral salts taken in small quantities very largely diluted, in imitation of natural mineral waters, have been found very useful. All measures likely to restore the functions of the stomach are to be put in requisition, and all flatulent and acidifying foods, spirituous and other exciting liquids, (such as tea and coffee,) religiously abstained from as long as there exists a marked excess of vascular action. At a later period of the disorder, however, a little mild wine has not appeared injurious. Gentle exercise and travelling are particularly recommended, together with the avoidance of everything which exhausts the strength and spirits, or ruffles the temper.

It will thus be seen that Dr. Hohnbaum's views of this affection coincide in many points with those more recently advocated by Mr. Faussett. (See No. IX. of this Journal.) But whilst they both agree as to the co-existence of derangement of the digestive organs, Mr. F. ventures a step beyond our more cautious German, and places the essence of the disorder in epigastric congestion or chronic inflammation, implicating the *ganglionic nerves and plexuses* and thence reflected on the aorta. He believes that there is in every case a considerable fulness, occasionally amounting to tumour, in the epigastric region, together with pain on pressure. In opposition to Dr. Hohnbaum as well as to Dr. Baillie, he thinks the affection is more common in women than in men. He takes a more serious view of the prognosis and employs the antiphlogistic treatment, (including the use of mercury and counter-irritation,) with greater activity and perseverance.

---

